# Acute pericarditis following treatment of a metastatic liver tumor with radiofrequency ablation: a case report

**DOI:** 10.1186/s12872-018-0937-7

**Published:** 2018-10-22

**Authors:** Jung-Chi Hsu, Han-Lin Tsai, Yu-Ling Lin, Rei-Yeuh Chang

**Affiliations:** 1 Division of Cardiology, Department of Internal Medicine, Ditmanson Medical Foundation Chiayi Christian Hospital, 539 Jhongsiao Road, Chia-Yi, 60002 Taiwan; 2 Graduate Institute of Clinical Medicine, National Taiwan University College of Medicine, Taipei, Taiwan; 3 Division of Gastroenterology, Department of Internal Medicine, Ditmanson Medical Foundation Chiayi Christian Hospital, Chiayi, Taiwan; 4 Department of Nursing, Chung Jen Junior College of Nursing, Health Sciences and Management, Chiayi, Taiwan; 5 Department of Beauty and Health Care, Min-Hwei Junior College of Health Care Management, Tainan, Taiwan

**Keywords:** Liver tumor, Radiofrequency ablation, Pericarditis, Case report

## Abstract

**Background:**

Radiofrequency ablation is a common and minimally invasive procedure used to treat liver tumors. However, the potential threat of heat injury to adjacent structures if the hepatic lesion is near the diaphragm is often overlooked and misunderstood. Rare cardiovascular complications have been reported. How best to identify the patients at risk to allow for prompt treatment is an important issue.

**Case presentation:**

A 56-year-old man with underlying oral cancer received radiofrequency ablation for a metastatic liver tumor at segment II. Pleuritic chest pain developed on the day after radiofrequency catheter ablation. Diffuse ST elevation and echocardiography showed the new onset of small to moderate pericardial effusion without tamponade sign. Inflammatory markers were also elevated. Acute pericarditis due to heat penetration and stimulation was favored. His symptoms and signs resolved after treatment with anti-inflammatory medication.

**Conclusion:**

Potential cardiovascular complications are possible after radiofrequency catheter ablation for liver tumors located at segment II. Artificial ascites with normal saline before radiofrequency ablation may separate the liver and diaphragm to prevent cardiac complications. During the procedure, electrocardiographic monitoring and close observation of the patient’s symptom are required. Echocardiography can be used to confirm cardiac complications.

## Background

Radiofrequency ablation (RFA) is a common procedure used to treat both primary and metastatic liver tumors in patients who are not going to receive surgical treatment [1, 2]. Major complications following RFA treatment for liver tumors include liver failure, hemorrhage, bile duct injury, abscess formation and colon perforation. Minor complications include fever, ascites, abdominal pain and thrombocytopenia [1]. Herein, we report the first case of pericarditis after RFA for metastatic liver tumors.

## Case presentation

**Fig. 1:**
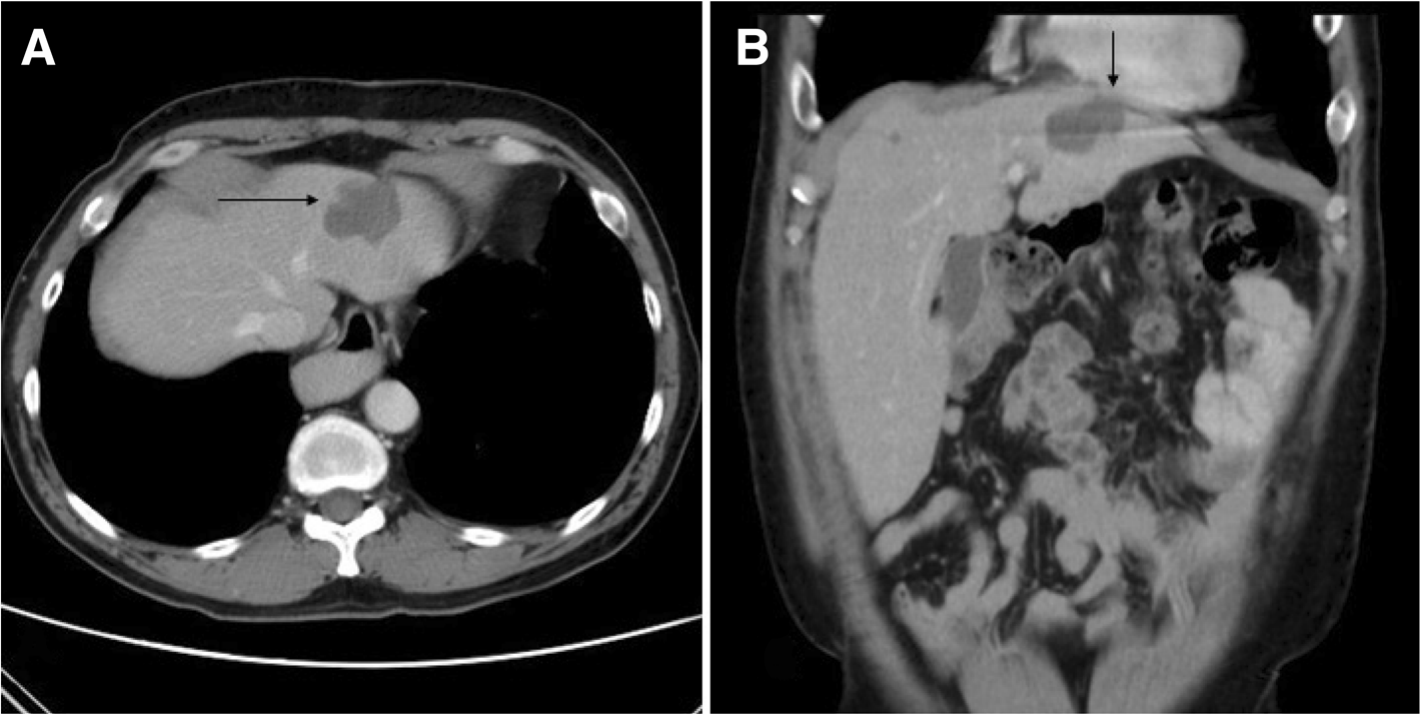
Abdominal computed tomography scan showed a 3.6 × 2.2 cm liver tumor in S2 segment of the left lobe of the liver (right arrow, **a**) in axial view and adjacent to the parietal pericardium of the inferior wall of the left ventricle in coronary view (down arrow, **b**)

A 56-year-old male patient with a history of paroxysmal atrial fibrillation was diagnosed with hypopharyngeal squamous cell carcinoma in June 2011. The disease progressed with pathology-proven metastatic liver tumor, for which he first received RFA in October 2012. In June 2014, abdominal sonography disclosed a mixed echogenic tumor of about 5 × 4 × 4 cm located in the left lobe just below the heart. A computed tomography scan showed a recurrent liver tumor of around 3.6 cm in the S2 segment (Fig. 1). He was admitted for repeat RFA on August 5th, 2014. Three Covidien 15 × 3 cm cool-tip needles were inserted under echo guidance, and RFA was smoothly applied for a total of 16 min. No immediate complications were noted during the procedure.

**Fig. 2:**
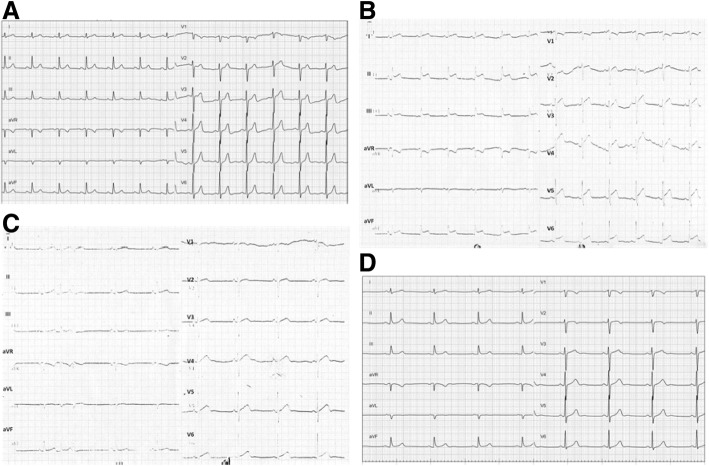
Serial electrocardiography recordings showed a normal sinus rhythm without ST segment elevation before radiofrequency ablation (**a**). Diffuse ST segment elevation except for lead V1 and aVR during chest pain (**b**). ST segment elevation returned to normal after 3 days (**c**) and 1 month (**d**) after the onset of chest pain

**Fig. 3:**
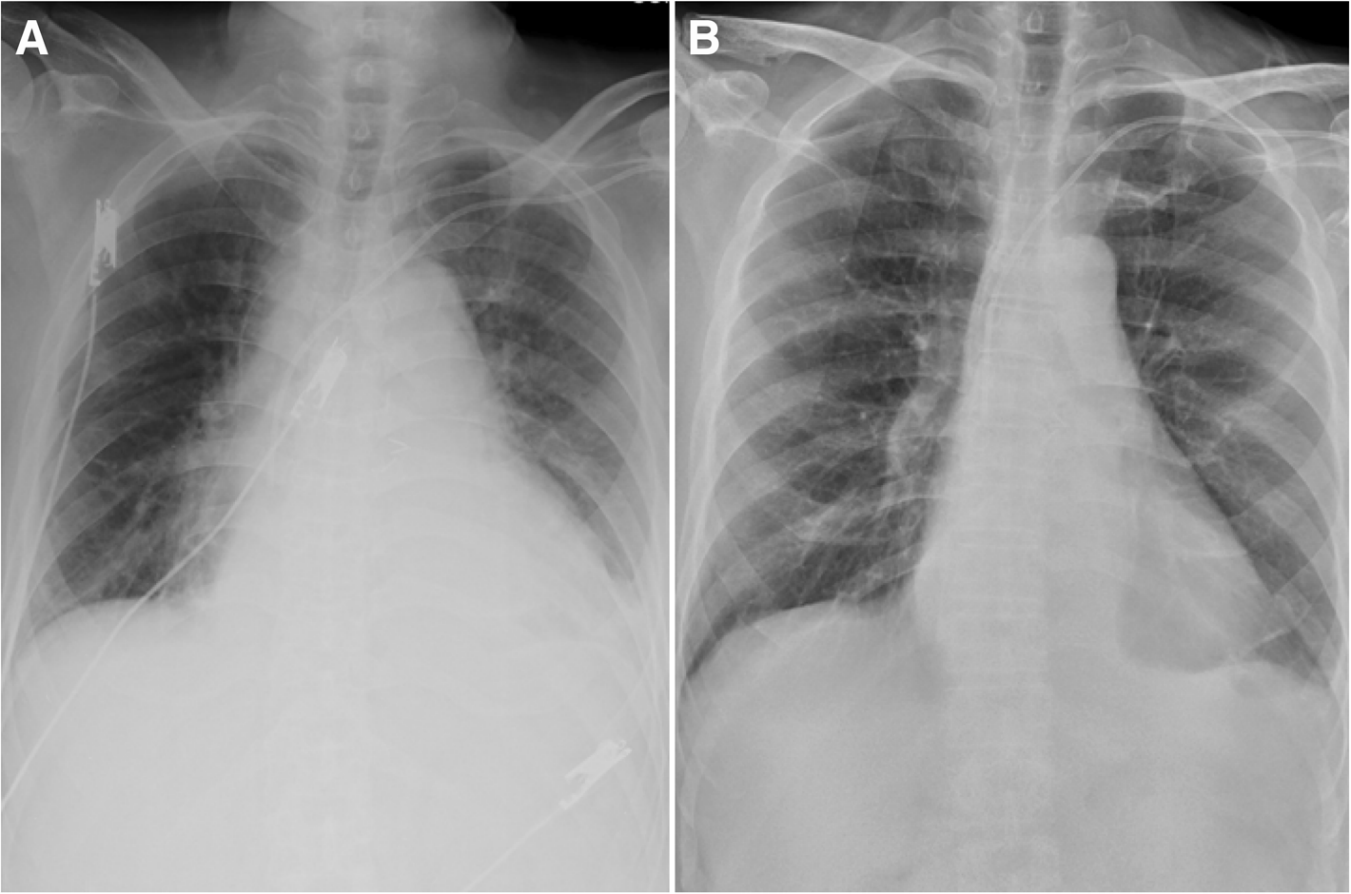
Chest x-ray showing cardiomegaly and left pleural effusion during chest pain (**a**) and decreased heart size and left pleural effusion 3 days later (**b**)

**Fig. 4:**
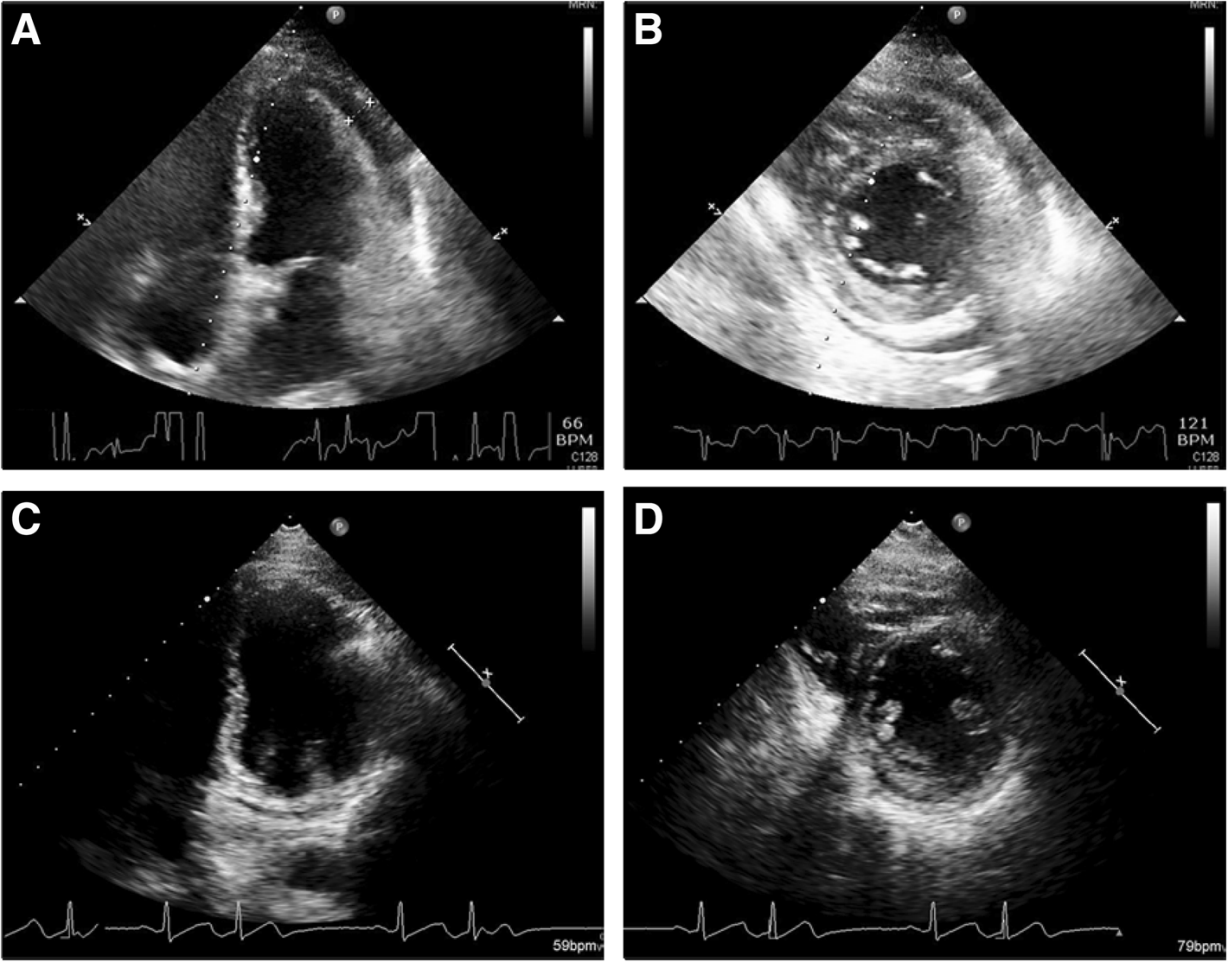
Echocardiography showing a small amount of pericardial effusion about 1.12 cm (arrow) during chest pain on parasternal long axis view (**a**) and short axis view (**b**). No pericardial effusion on parasternal long axis view (**c**) and short axis view 1 month later (**d**)

Upper abdominal fullness was noted after the RFA. Abdominal sonography showed no new ascites and his hemogram remained stable. Hemorrhagic complications were therefore unlikely. The abdominal pain and fullness gradually subsided on the next day, however new-onset chest pain and paroxysmal atrial fibrillation with a rapid ventricular response developed. Fever and chills were also noted, and a physical examination revealed friction rub. An electrocardiogram (ECG) showed diffuse ST elevation except for leads AVR and V1 (Fig. 2b), and the baseline ECG was normal (Fig. 2a). A chest X-ray showed a water-drop appearance and left pleural effusion (Fig. 3a). Inflammatory markers including leukocyte count and the concentration of serum C-reactive protein (CRP) were elevated (Hb 9.6 g/dL, WBC 10300/L, CRP 19.8 mg/dL), however levels of cardiac enzymes were not elevated (CK 202 U/L, CK-MB 0.6 ng/mL, troponin-I 0.013 ng/mL). Echocardiography showed a small amount of pericardial effusion with a maximal depth of about 1.12 cm, without signs of tamponade (Fig. 4a, b). Acute pericarditis was the most likely diagnosis, and he was treated with aspirin 100 mg and diclofenac 100 mg per day. Three days later, the ST segment (elevations) had mostly returned to baseline (Fig. 2c) and the size of his heart and left pleural effusion had also decreased (Fig. 3b). His symptoms improved and the levels of inflammatory markers declined (Hb 9.1 g/dL, WBC 7720/L, CRP 8.357 mg/dL). He was discharged uneventfully five days later. There was no pericardial effusion (Fig. 4c, d) and the ST segments had totally returned to baseline (Fig. 2d) 1 month later.

## Discussion and Conclusion

Pericarditis is the most common form of pericardial disease with causes including idiopathic, infectious (e.g. viral, bacterial), systemic inflammatory diseases (e.g. systemic lupus erythematosus, systemic vasculitis, sarcoidosis, inflammatory bowel disease), cancer, metabolic (e.g. uremia, myxedema, anorexia nervosa), drugs (e.g. interferon, doxorubicin, cyclophosphamide, penicillins), traumatic, iatrogenic, and post-cardiac injury syndrome. The diagnosis is based on clinical criteria including chest pain, pericardial rub, ECG changes, and pericardial effusion [3].

A few case reports have indicated that delayed tamponade with Dressler’s syndrome can occur after radiofrequency catheter ablation to isolate the pulmonary vein for atrial fibrillation or after radiofrequency catheter ablation for atrial flutter, AV node, or accessory pathway [4–7]. In addition, Liu et al. reported that 83% of their patients with post-cardiac injury syndrome had pleural effusion [8]. Apart from ablation for arrhythmias, several case reports have described acute pericarditis after percutaneous coronary interventions [9, 10]. A pericardial inflammatory reaction may be the cause of direct cardiac injury rather than perforation, however it could also result from an indirect cardiac injury such as a non-penetrating thoracic injury or radiation injury.

Pericarditis or cardiac tamponade after RFA for a liver tumor is also a rare complication. Allgaier et al. treated 12 consecutive patients with inoperable hepatic carcinoma with radiofrequency interstitial thermal ablation in 1998, and considered it to be an effective and safe palliative therapeutic strategy [1]. Since then, RFA has gradually become one of the primary methods used for the local treatment of liver tumors. RFA is also used to treat tumors close to large blood vessels in the liver or complex foci in extrahepatic tissues [2]. The main complications of RFA include fever, pain, abdominal bleeding, bile duct injury, bowel injury, liver abscesses and implantation metastasis. There were no previous reports of pericardial effusion before 2005.

Cardiac hemorrhagic tamponade during RFA is an extremely uncommon but potentially fatal complication [11]. Silverman et al. reported that RFA for a liver tumor in segment II of the liver increased the risk of hemorrhagic tamponade [12], and Moumouh et al. reported the first case of cardiac tamponade during RFA leading to death in 2005 [13]. Gao et al. reported a similar case with acute hemorrhagic tamponade who was successfully treated in 2010 [14]. The liver tumors in all of these cases were located at lateral segment II, and even with the use of a fine needle for laser thermal ablation, hemorrhagic cardiac tamponade still occurred. A possible explanation for cardiac tamponade is that it may be difficult to ascertain the exact location of the ablation needle even with an experienced operator and echo/computed tomography guidance. In such cases, direct injury of the anterior cardiac vein or microperforations of vessels can occur. Another possibility is that the distribution of heat in vivo may be unpredictable, which may lead to injury of the pericardium through heat conduction.

In addition to hemorrhagic tamponade, small pericardial effusion can occur due to the adjacent tissues and organs being damaged during RFA as the result of pericarditis. For tumors located at the liver capsule on the left hepatophrenic side close to the diaphragm, the thermal conduction during RFA may cause damage to the diaphragm and pericardium. This may then lead to localized pericardial edema causing compression of the suprahepatic vena cava, and eventually resulting in pericardial effusion [15].

In our case, similar to the previous cases, the liver tumor was at segment II. The symptoms and signs of pericarditis may have been masked by the fever, chills and leukocytosis, all of which can occur after RFA for a liver tumor. However, the abrupt elevation of serum C-reactive protein level and the widespread ST elevation with new onset of pericardial effusion strongly supported the diagnosis of acute pericarditis. Since the clinical course was relatively benign, the mechanism may have been the result of pericardial/myocardium damage due to heat injury through the diaphragm and indirect inflammatory reactions rather than vessel perforation. The complication of pericarditis or left pleural effusion may be predicable and should be carefully considered before the procedure if the hepatic lesion is at segment II. Artificial ascites with normal saline before RFA may separate the liver and diaphragm to prevent cardiac complications. The procedure should be conducted under echo or computed tomography guidance to confirm the location of the tumor. During the procedure, electrocardiographic monitoring and close observation of the patient’s symptom are required. The procedure should be stopped if arrhythmia or hemodynamic changes occur. Echocardiography can be used to confirm cardiac complications.

In summary, we present a rare complication of acute pericarditis following RFA for a liver tumor in whom the symptoms and ECG almost resolved. Adequate timely management is important in such cases. Although the minimally invasive RFA has become a preferred approach with an increasingly significant role in the field of multidisciplinary comprehensive treatment for liver tumors, the possibility that adjacent tissues and organs may be damaged during treatment still exists. Clinicians should be fully aware of all potential complications related to RFA. Timely recognition and prompt management of pericarditis and pericardial effusion are important, and the prognosis is relatively good if tamponade does not occur.

## Abbreviations

CK: Creatine kinase
CK-MB: Creatine kinase MB isoenzyme
CRP: C-Reactive protein
ECG: Electrocardiogram
RFA: Radiofrequency ablation
WBC: White blood cell
